# Causes and extent of avoidable mortality across the european union: insights for advancing healthy aging

**DOI:** 10.1007/s11357-025-02045-2

**Published:** 2026-02-13

**Authors:** David Major, Vince Fazekas-Pongor, Nóra Kovács, Péter Pikó, Mónika Fekete, Zoltan Ungvari, Róza Ádány

**Affiliations:** 1 Institute of Preventive Medicine and Public Health, Semmelweis University, Budapest, Hungary; 2 Fodor Center for Prevention and Healthy Aging, Semmelweis University, Budapest, Hungary; 3 Outpatient Clinic, Semmelweis University, Budapest, Hungary; 4 Department of Public Health and Epidemiology, Faculty of Medicine, University of Debrecen, Debrecen, Hungary; 5 Department of Public Health and Epidemiology, Faculty of Medicine, HUN-REN-UD Public Health Research Group, University of Debrecen, Debrecen, Hungary; 6 Center for Epidemiology and Surveillance, National Laboratory for Health Security, Semmelweis University, Budapest, Hungary; 7 Vascular Cognitive Impairment, Neurodegeneration and Healthy Brain Aging Program, Department of Neurosurgery, University of Oklahoma Health Sciences Center, Oklahoma City, OK USA; 8 Doctoral College/Institute of Preventive Medicine and Public Health, International Training Program in Geroscience, Semmelweis University, Budapest, Hungary; 9 Department of Health Promotion Sciences, College of Public Health, University of Oklahoma Health Sciences Center, Oklahoma City, OK USA

**Keywords:** Avoidable mortality, Preventable mortality, Treatable mortality, Aging, Cluster analysis, Public health

## Abstract

Although population aging is a global worldwide phenomenon, it is most pronounced in European Union countries. An unhealthy ageing population not only places a heavy burden on healthcare, but also hinders sustainable socio-economic development. Supporting healthy aging can be achieved through evidence-based strategies aimed at reducing avoidable mortality and interventions that promote these strategies. Our study is based on a secondary analysis of Eurostat data for 2022 to describe the extent and structure of avoidable (divided to preventable and treatable) mortality stratified by sex for each EU country. Cluster analyses based on avoidable mortality indicators (preventable mortality rate, treatable mortality rate, preventable-to-treatable mortality ratio, and male-to-female avoidable mortality rate ratio) and the cause-specific composition of avoidable mortality were used to highlight similarities and differences between countries. Classification based on the available mortality indicators revealed regional patterns. Western and Northern EU countries were predominantly in Cluster 1 which had the lowest preventable and treatable mortality rates, as well as relatively balanced sex ratios. However, these countries had a higher preventable-to-treatable mortality ratio indicating potential delays or gaps in preventive services. Mediterranean EU countries were in Cluster 2 showing moderate values for the avoidable mortality indicators and relatively low preventable-to-treatable mortality ratios. However, the male-to-female mortality ratio was high, indicating significant sex disparities. Central and Eastern-European EU countries were in Cluster 3 which had the highest burden of both preventable and treatable mortality. The preventable-to-treatable mortality ratio was lower than in Cluster 1, and the male-to-female mortality ratio was the highest in this cluster. Cluster analysis based on the cause-specific composition of avoidable mortality also identified three clusters with leading mortality causes as (1) cardiovascular diseases mainly in Clusters 2 and 3 countries; (2) cancer dominantly for Cluster 1 countries; and (3) high proportions of cardiovascular and alcohol-related mortality in mainly Cluster 3 countries. The clusters do not align perfectly (Adjusted Rand Index = 0.240, Normalized Mutual Information = 0.337), the two approaches complement each other. The first approach identified countries with similar levels and patterns in terms of preventable and treatable types of avoidable mortality, while the second approach highlighted similarities in the composition of health threats. Combining the two approaches offers a more detailed understanding of regional health profiles within the European Union, and enables the development of targeted interventions to reduce avoidable mortality and promote healthy aging.

## Introduction

Europe’s population is aging rapidly [1] due to declining fertility rates and rising life expectancy. In 2022, over 20% of EU-27 residents were aged 65 or older, up from 16% in 2000, and this share is projected to reach nearly 30% by 2050 [2]. Although life expectancy continues to rise, healthy life years have not increased at the same rate [3]. Between 2000 and 2022, life expectancy increased by nearly 8.2 years (from around 72.4 years in 2000 to 80.6 years in 2022), but healthy life expectancy saw a more modest increase, rising by approximately 4.5 years (from 58.1 years in 2000 to 62.6 years in 2022) [4].

Parallel, the prevalence of age-related diseases [5] such as cardiovascular morbidities, cancer, and diabetes mellitus continues to rise leaving many individuals in poor health in a significant portion of their later years. Diseases of the circulatory system accounted for 32.7% deaths in the EU, followed by cancer (22.3%) and respiratory diseases in 2022 [6]. As a result, aging societies face growing demands for both preventive and curative health services, along with escalating healthcare costs [7]. Understanding how to extend not just lifespan, but healthspan, is therefore a key priority from both a public health and an economic point of view [8].

Avoidable mortality remains a critical indicator of health system performance and public health effectiveness [9]. Defined as deaths that could have been prevented through effective public health interventions or treated with timely and high-quality healthcare [10], avoidable mortality reflects both population-level risk exposure and the capacity of health services to detect diseases early and provide effective treatment. These categories (preventable and treatable mortality) offer complementary perspectives on where interventions can most effectively reduce premature deaths.

The present paper is particularly pressing in the context of Europe's aging population [11]. In many countries, the burden of chronic diseases and associated healthcare costs is growing [12]. Furthermore, individuals who die prematurely from avoidable causes may never reach older age, thereby undermining efforts to promote healthy aging [13, 14].

Although countries within the EU operate under shared policy goals, yet show significant variation in mortality outcomes, healthcare investment, and effectiveness of public health interventions [15–17]. There is an eight‑year life expectancy gap between countries with the highest and lowest life expectancies [2]. Furthermore, despite the fact that avoidable cardiovascular mortality has declined by more than 50% since the mid-1990s, improvement has been uneven across diseases, demographic groups, and regions [13]. Socioeconomic inequalities in education, income, and healthcare access further shape these patterns [18].

Analyzing avoidable mortality serves two purposes. Firstly, it highlights the gaps in the prevention and treatment of diseases that can be cured if diagnosed and treated in time. Secondly, it identifies the systemic weaknesses that could hinder future health improvements, especially in aging populations. Despite extensive reporting on preventable and treatable mortality, no EU-wide analysis has yet integrated both the overall levels and the cause-specific composition of avoidable deaths using the harmonized, post-pandemic 2022 Eurostat dataset. It remains unknown whether countries that appear similar in their balance of preventable and treatable mortality also share the same dominant causes of avoidable death, or whether different underlying threats drive similar aggregate outcomes. Identifying patterns and clusters of countries with similar avoidable mortality profiles can inform more effective, tailored health policy responses.

The aim of this study is to examine avoidable mortality in EU countries using the most recent Eurostat data. Two cluster analyses were conducted to explore distinct patterns in (1) overall levels and ratios of preventable and treatable mortality, and (2) the cause-specific composition of avoidable deaths. The year 2022 offers a particularly relevant snapshot, as it reflects the post-pandemic recovery of health systems and the lingering effects of service disruptions, delayed diagnoses, and exacerbated inequalities [19–22]. The findings could inform future strategies aimed at reducing avoidable deaths and promoting healthy aging across Europe [23].

## Methods

### Data source

This study is a secondary analysis on publicly available data retrieved from the Eurostat online database (https://ec.europa.eu/eurostat) on avoidable mortality in the European Union (EU) for the year 2022 [24]. The dataset includes information reported by national statistical offices and harmonized by Eurostat in accordance with international standards. Data were downloaded in May 2025, and no additional data cleaning, or transformation was performed.

### Definition of avoidable mortality

The concept of avoidable mortality used in this report follows Eurostat’s operational classification, which distinguishes: (1) Preventable deaths: those that could have been avoided through effective public health and primary prevention strategies; (2) Treatable (amenable) deaths: those that could have been avoided through timely and appropriate healthcare interventions; (3) Total avoidable mortality: the combination of preventable and treatable deaths. Causes of death were classified based on the OECD/Eurostat list of avoidable mortality, which uses ICD-10 codes to identify relevant conditions. In cases where there was no clear evidence of the predominance of preventability or treatability (e.g. ischaemic heart disease, stroke and diabetes), the causes were divided equally between the two categories to avoid double counting the same cause of death in both lists. The age threshold for premature (avoidable) mortality is 74 years for all causes [10].

### Variables and indicators

The primary indicators analyzed were the age-standardized avoidable, preventable and treatable mortality rates per 100,000 population, standardized to the 2013 European Standard Population. COVID-19-related deaths (ICD-10 U07.1- U07.2) were removed to ensure that variations in avoidable mortality were not confounded by the uneven impact of the pandemic across member states. Data were reported by EU member state (EU-27), sex (male, female), category (preventable, treatable, avoidable) and leading causes (cancer, cardiovascular diseases, injuries, alcohol- and drug related deaths, respiratory diseases, endocrine and metabolic diseases, others – based on OECD/Eurostat list [10]).

### Statistical analysis

Tables and figures were created to summarize and illustrate avoidable, treatable and preventable mortality rates by country, differences between male and female avoidable mortality rates, and leading causes of avoidable mortality in EU member states.

To explore patterns in avoidable mortality profiles across the European Union, two complementary cluster analyses were performed. The first one aimed to reflect the magnitude and structural balance of avoidable mortality burdens. Four standardized variables were used: (1) the age-standardized preventable mortality rate, (2) the age-standardized treatable mortality rate, (3) the preventable-to-treatable mortality ratio, and (4) the male-to-female avoidable mortality rate ratio. Each variable was standardized (z-scores) prior to analysis to ensure comparability. The second analysis used the compositional distribution of causes of avoidable mortality, providing insight into the underlying epidemiological profile of each country. Variables used in the second cluster analysis were the percentages of the various subcategories (cancer, cardiovascular diseases, injuries, alcohol- and drug related deaths, respiratory diseases, endocrine and metabolic diseases, others) of avoidable mortality within each member state. Hierarchical cluster analyses using Ward’s method and squared Euclidean distance were employed to identify groups of countries with similar avoidable mortality profiles. The number of clusters was determined by visually inspecting the dendrograms and the agglomeration schedules. A marked increase in the fusion coefficient at the final stage supported a three-cluster solution for both analyses. Between-group differences were assessed with Kruskal–Wallis H test. Pairwise post-hoc comparisons were performed using the Mann–Whitney U test with Bonferroni correction. The cluster maps were generated in Flourish Studio (https://app.flourish.studio/). Finally, Adjusted Rand Index (ARI) and Normalized Mutual Information (NMI) were calculated to measure similarity between the two clusterings. All statistical analyses were performed using IBM SPSS Statistics version 30.0.0.0, except for ARI and NMI which was calculated in STATA version 17.0.

## Results

### Avoidable, preventable and treatable mortality by country

**Fig. 1:**
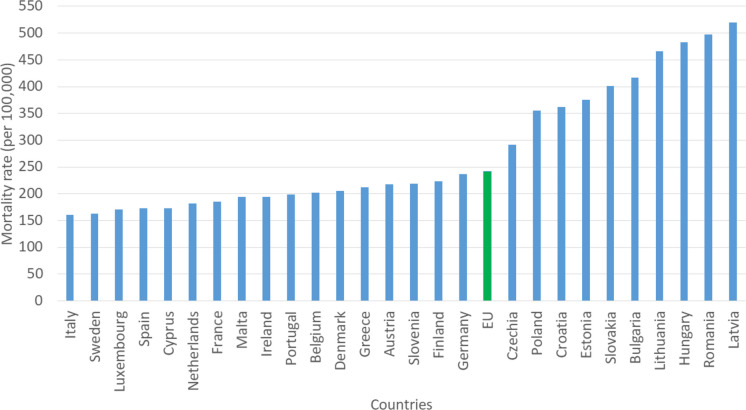
Avoidable mortality rates (without COVID-19 related deaths) in the EU by country in 2022

The total avoidable mortality rate in the EU was 241.3 per 100,000 people excluding deaths related to COVID-19 pandemic in 2022. Italy, Sweden and Luxembourg had the lowest rates with 160.7, 163.0, and 170.9 per 100,000 people, respectively. The countries with the highest rates were Latvia, Romania, and Hungary experiencing rates approximately double of the EU average: 519.7, 467.7, and 483.3 per 100,000 people respectively. Figure 1 demonstrates the avoidable mortality rates by country.

**Fig. 2:**
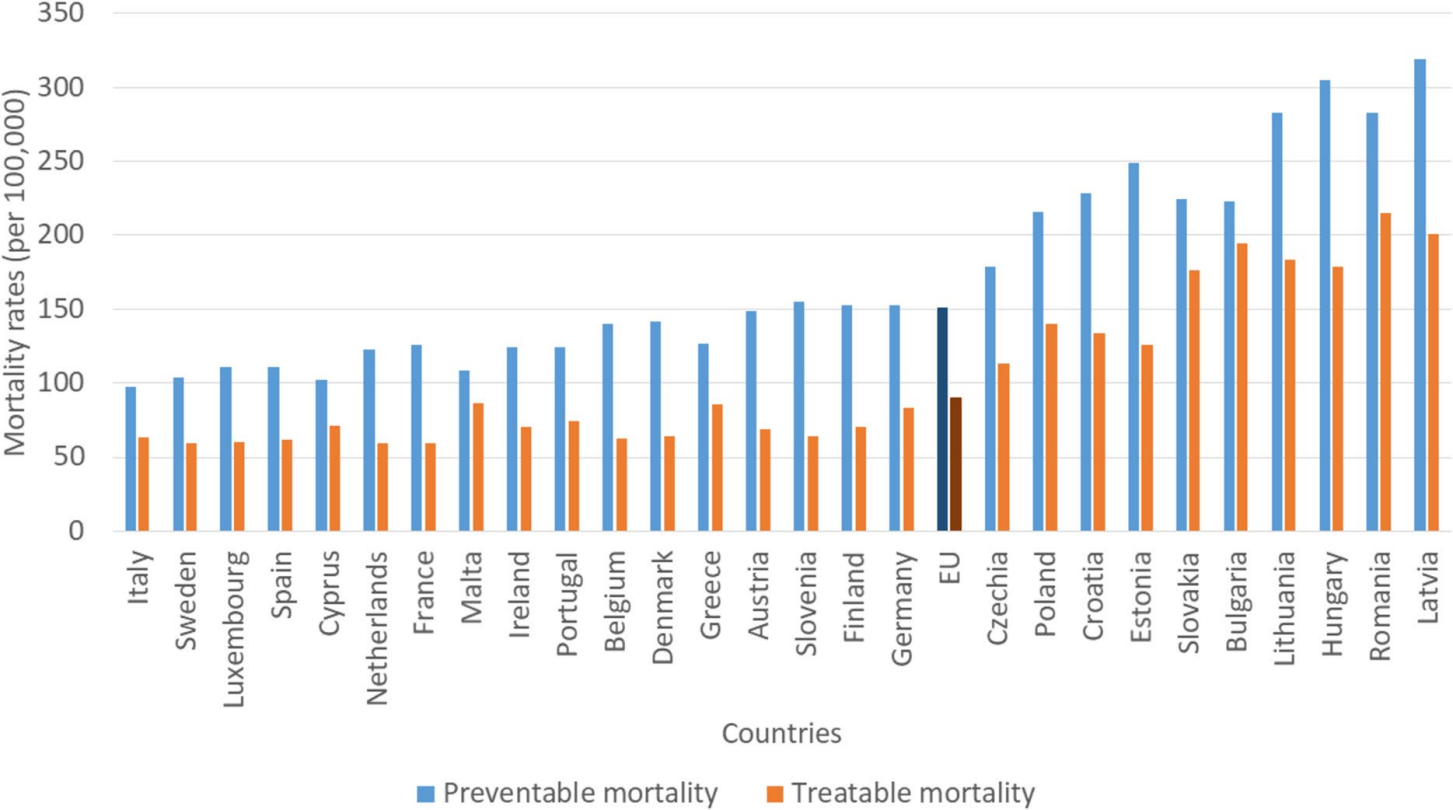
Preventable (without COVID-19 related deaths) and treatable mortality rates in the EU by country in 2022

Figure 2 demonstrates preventable and treatable mortality rates by country. Total preventable mortality rate in the EU was 151.6 per 100,000 people excluding deaths related to COVID-19 in 2022. Italy, Cyprus, and Sweden experienced the lowest rates of preventable deaths with 97.3, 102.4, and 103.8 per 100,000 people respectively. The highest rates occurred in Latvia, Hungary, and Lithuania with approximately double of the EU average again: 319.0, 304.9, and 238.1 per 100,000 people respectively. The average treatable mortality rate in the EU was 89.7 per 100,000 people. The countries with the lowest mortality rates were Sweden, the Netherlands, and France with 59.2, 59.2, and 59.4 per 100,000 people, respectively. Romania, Latvia, and Bulgaria had the highest treatable mortality rates at 215.0, 200.7, and 194.1 per 100,000 people respectively, more than twice the EU average. Preventable mortality rates were higher than treatable mortality rates in all countries. The ratio of preventable-to-treatable mortality rates varied across the EU: Bulgaria, Malta, and Slovakia had relatively similar rates of preventable and treatable mortality (ratios of 1.15, 1.26, 1.27, respectively), while Slovenia, Belgium, and Denmark experienced the greatest relative difference (ratios of 2.42, 2.25, 2.21, respectively).

### Sex differences in avoidable mortality

**Fig. 3:**
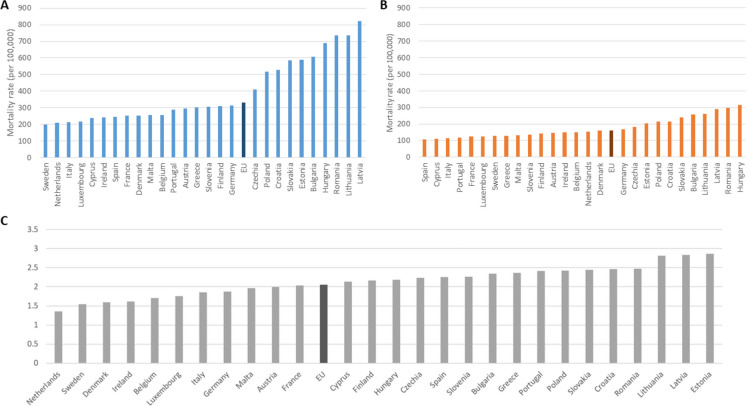
Avoidable mortality rates among males (**A**), females (**B**), and male-to-female ratio of avoidable mortality rates (**C**) in the EU by country in 2022

Average avoidable mortality rates in the EU for males and females were 329.8 and 160.3 per 100,000 people, meaning that males experienced a 2.06 higher avoidable mortality rate (Fig. 3). Sweden, the Netherlands, and Italy showed the lowest avoidable mortality rates for males: 198.1, 209.7, and 211.1 per 100,000 people, respectively. The countries with the highest rates for males were Latvia, Lithuania, and Romania having approximately four times higher rates than the previous countries: 822.1, 736.4, and 734.2 per 100,000 people, respectively. For females, Spain, Cyprus, and Italy had the lowest rates of avoidable mortality with 107.9, 111.9, and 114.1 per 100,000 people respectively. The highest avoidable mortality rates occurred in Hungary, Romania, and Latvia: 315.2, 297.2, and 289.9 per 100,000 people. The avoidable mortality rate was consistently higher for males than for females in all EU countries. The smallest inequalities were experienced in the Netherlands, Sweden, and Denmark (ratios: 1.35, 1.55, 1.59, respectively), while the highest inequalities in Estonia, Latvia, and Lithuania (ratios: 2.86, 2.84, 2.82, respectively).

### Main causes of avoidable mortality

**Fig. 4:**
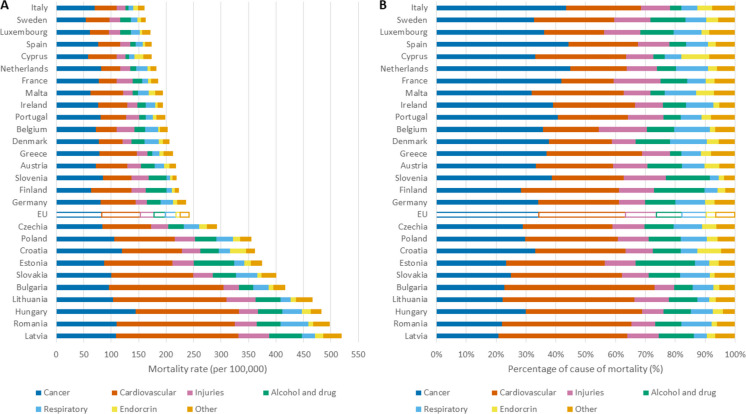
Causes of avoidable mortality in rate (**A**) and in percentages (**B**) in the EU by country in 2022

The most common cause of avoidable mortality was cancer (82.8 per 100,000 people; 34.3%) in the EU in 2022. It was followed by cardiovascular diseases (69.8 per 100,000 people; 28.9%), injuries (24.9 per 100,000 people; 10.3%), alcohol- and drug-related deaths (21.0 per 100,000 people; 8.7%), respiratory diseases (19.5 per 100,000 people; 8.1%), and endocrine diseases (7.9 per 100,000 people; 3.3%). However, this order was not consistent across all EU member states. Cardiovascular disease was the most common cause of avoidable mortality in Bulgaria, the Czechia, Estonia, Finland, Hungary, Latvia, Lithuania, Poland, Romania, and Slovakia (Fig. 4).

**Fig. 5:**
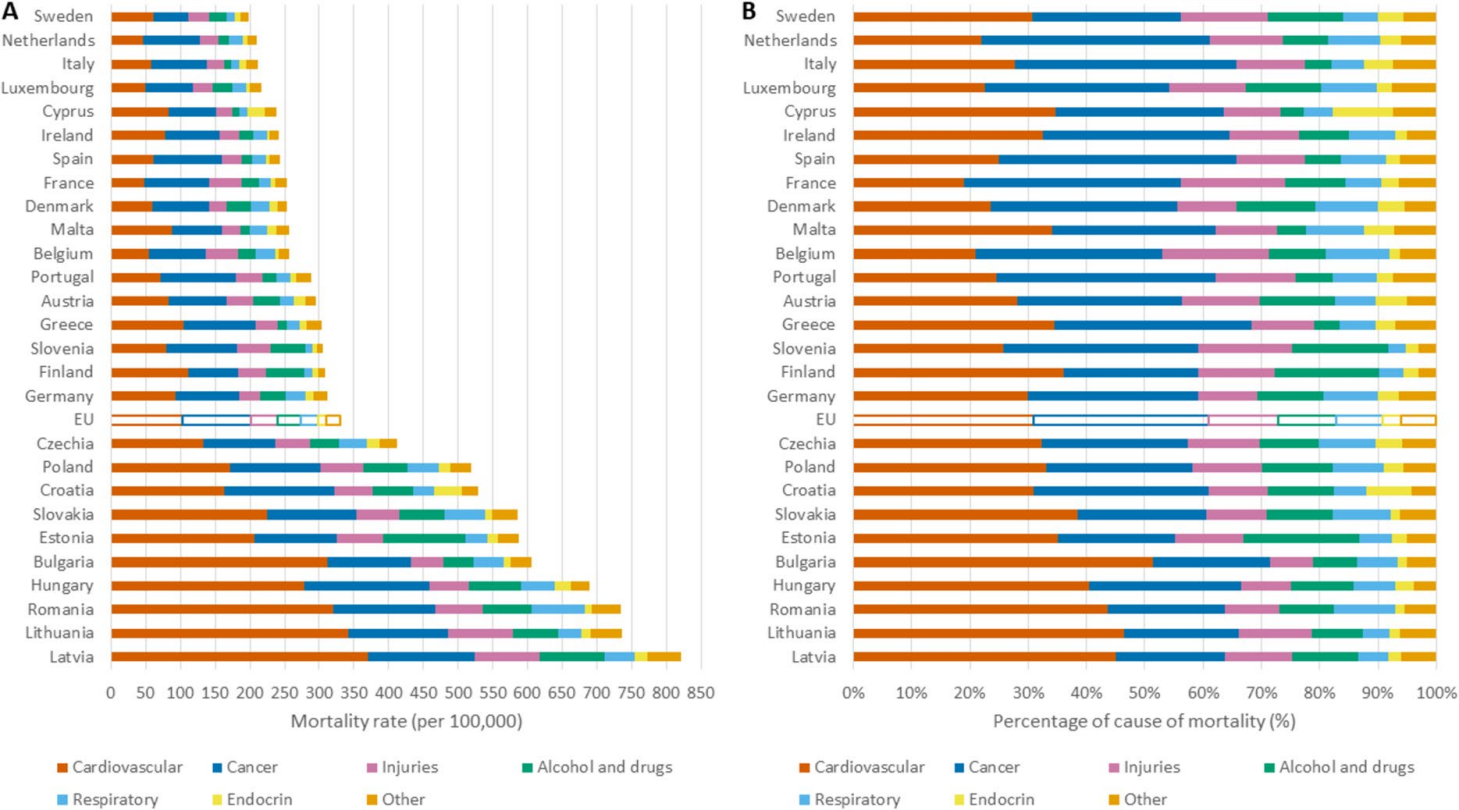
Causes of avoidable mortality in rate (**A**) and in percentages (**B**) in the EU by country in 2022 among males

Sex specific differences could be observed in main causes of avoidable mortality. The most common cause was cardiovascular diseases (102.2 per 100,000 people) in the EU in 2022 among males. Cancer was the second most common cause with 98.9 per 100,000 people. Other causes followed the same order as in the total population (injuries: 39.4 per 100,000; alcohol and drugs: 32.3 per 100,000; respiratory: 26.2 per 100,000; endocrine: 10.8 per 100,000). Nevertheless, in approximately one-third of countries (Austria, Belgium, Denmark, France, Italy, Luxembourg, the Netherlands, Portugal, Slovenia, and Spain), cancer accounted for the largest share of cause-specific avoidable mortality among males (Fig. 5).

**Fig. 6:**
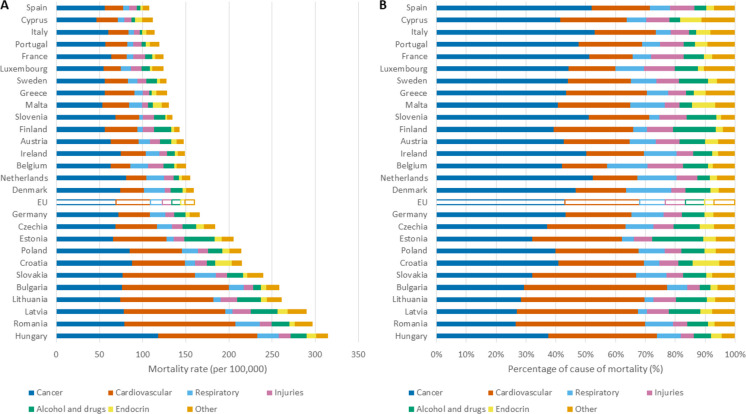
Causes of avoidable mortality in rate (**A**) and in percentages (**B**) in the EU by country in 2022 among females

As for females, the most common cause of avoidable mortality was cancer (68.9 per 100,000 people) followed by cardiovascular diseases (40.4 per 100,000 people) in the EU. Cardiovascular diseases accounted for the largest share only in Bulgaria, Hungary, Latvia, Lithuania, and Romania. Respiratory diseases occurred as the third most common cause in the EU (13.5 per 100,000 people) followed by injuries (10.9 per 100,000 people), alcohol and drugs (10.3 per 100,000 people) and endocrine diseases (5.2 per 100,000 people) (Fig. 6).

### Cluster analysis based on avoidable mortality indicators

Using four standardized indicators (preventable mortality rate, treatable mortality rate, preventable-to-treatable mortality ratio, and male-to-female avoidable mortality rate ratio) a three-cluster solution was identified among EU member states. Characteristics of clusters are presented in Table 1. Cluster 1 included countries such as Austria, Belgium, Denmark, Finland, France, Germany, Ireland, Luxembourg, the Netherlands, Slovenia, and Sweden. They were characterized by lower preventable and treatable mortality rates compared to the other two groups, as well as relatively balanced sex ratios. This represented the most favorable profile in terms of avoidable mortality. However, they also represented countries with a higher preventable-to-treatable mortality ratio indicating potential delays or gaps in preventive services despite lower treatable mortality rates. Cluster 2 comprised Cyprus, Greece, Italy, Malta, Portugal, and Spain. They showed moderate values for the mortality indicators, with relatively low preventable-to-treatable mortality ratios. Their male-to-female mortality ratio was high, pointing to significant sex differences. Cluster 3 included Bulgaria, Croatia, Czechia, Estonia, Hungary, Latvia, Lithuania, Poland, Romania, and Slovakia. They showed the highest burden of both preventable and treatable mortality, reflecting significant health challenges. The preventable-to-treatable mortality ratio was lower than in Cluster 1, but male-to-female mortality gap was the highest in this cluster, highlighting marked sex inequalities in health. This classification also revealed regional patterns, with Western and Northern countries predominantly in Cluster 1, Mediterranean countries in Cluster 2, and Central and Eastern European (post-socialist) countries in Cluster 3.

**Table 1:** Characteristics of clusters based on avoidable mortality indicators

	Preventable mortality rate* (per 100,000 people)	Treatable mortality rate* (per 100,000 people)	Preventable-to-treatable ratio*	Male-to-female ratio*
Cluster 1 (Western/Northern)	134.4 (122.3—146.4)	65.7 (60.7—70.6)	2.05 (1.90—2.20)	1.81 (1.62—2.00)
Cluster 2 (Mediterranean)	111.6 (99.3—124.0)	73.8 (62.8—84.7)	1.53 (1.34—1.72)	2.16 (1.93—2.40)
Cluster 3 (Central/Eastern)	250.9 (218.9—282.9)	166.1 (141.0—191.1)	1.54 (1.37—1.71)	2.51 (2.33—2.68)
P-values of Kruskal–Wallis test	< 0.001	< 0.001	< 0.001	< 0.001
Pairwise comparisons (p-value of Mann–Whitney U^§^)				
C1 vs. C2	0.035	0.070	0.002	0.035
C1 vs. C3	< 0.001	< 0.001	< 0.001	< 0.001
C2 vs C3	0.001	0.001	0.664	0.017

*Data are presented as mean (95% confidence interval)

§Significance level adjusted to α = 0.017 using Bonferroni correction

### Cluster analysis based on the cause-specific composition of avoidable mortality

A second cluster analysis was conducted using the percentage distribution of causes of avoidable mortality. Details are presented in Table 2. Cluster 1 (Austria, Croatia, Cyprus, Czechia, Germany, Greece, Malta, Poland, Sweden) displayed a balanced distribution of cardiovascular and cancer-related avoidable mortality. They also experienced higher mortality of endocrine and metabolic causes compared to the other clusters. Cluster 2 (Belgium, Denmark, France, Ireland, Italy, Luxembourg, the Netherlands, Portugal, Slovenia, Spain) was characterized by high rates of cancer-related avoidable mortality, alongside relatively high rates of injury-related deaths. Cardiovascular mortality was comparatively low in this cluster. Cluster 3 (Bulgaria, Estonia, Finland, Hungary, Latvia, Lithuania, Romania, Slovakia) was characterized by high proportions of cardiovascular and alcohol-related mortality, which is indicative of structural health challenges and behavioral risk factors.

**Table 2:** Characteristics of clusters based on the cause-specific composition of avoidable mortality

	Cancer* (%)	Cardiovascular* (%)	Injuries* (%)	Alcohol and drugs* (%)	Respiratory* (%)	Endocrine* (%)	Other* (%)
Cluster 1	32.6 (30.8—34.4)	29.4 (27.7—31.1.)	10.0 (9.1—10.9)	8.4 (5.9—10.9)	7.8 (6.4—9.3)	5.2 (3.6—6.9)	6.5 (5.5—7.5)
Cluster 2	40.2 (37.9—42.6)	21.9 (19.6—24.2)	11.9 (9.9—13.8)	8.4 (6.1—10.8)	8.3 (5.7—10.6)	2.9 (2.2—3.6)	6.5 (5.4—7.6)
Cluster 3	24.3 (21.6—27.0)	40.3 (35.3—45.3)	9.4 (7.8—11.0)	11.5 (7.8—15.3)	6.5 (4.4—8.6)	2.4 (1.9—2.9)	5.5 (4.5—6.5)
P-values of Kruskal–Wallis test	** < 0.001**	** < 0.001**	0.132	0.331	0.353	** < 0.001**	0.356
Pairwise comparisons (p-values of Mann–Whitney U^§^)							
C1 vs. C2	**0.001**	** < 0.001**	0.501	0.386	0.211	** < 0.001**	0.248
C1 vs. C3	** < 0.001**	** < 0.001**	0.102	0.806	0.806	**0.003**	0.935
C2 vs C3	** < 0.001**	** < 0.001**	0.091	0.110	0.214	0.480	0.183

*Data are presented as mean (95% confidence interval)

§Significance level adjusted to α = 0.017 using Bonferroni correction

**Fig. 7:**
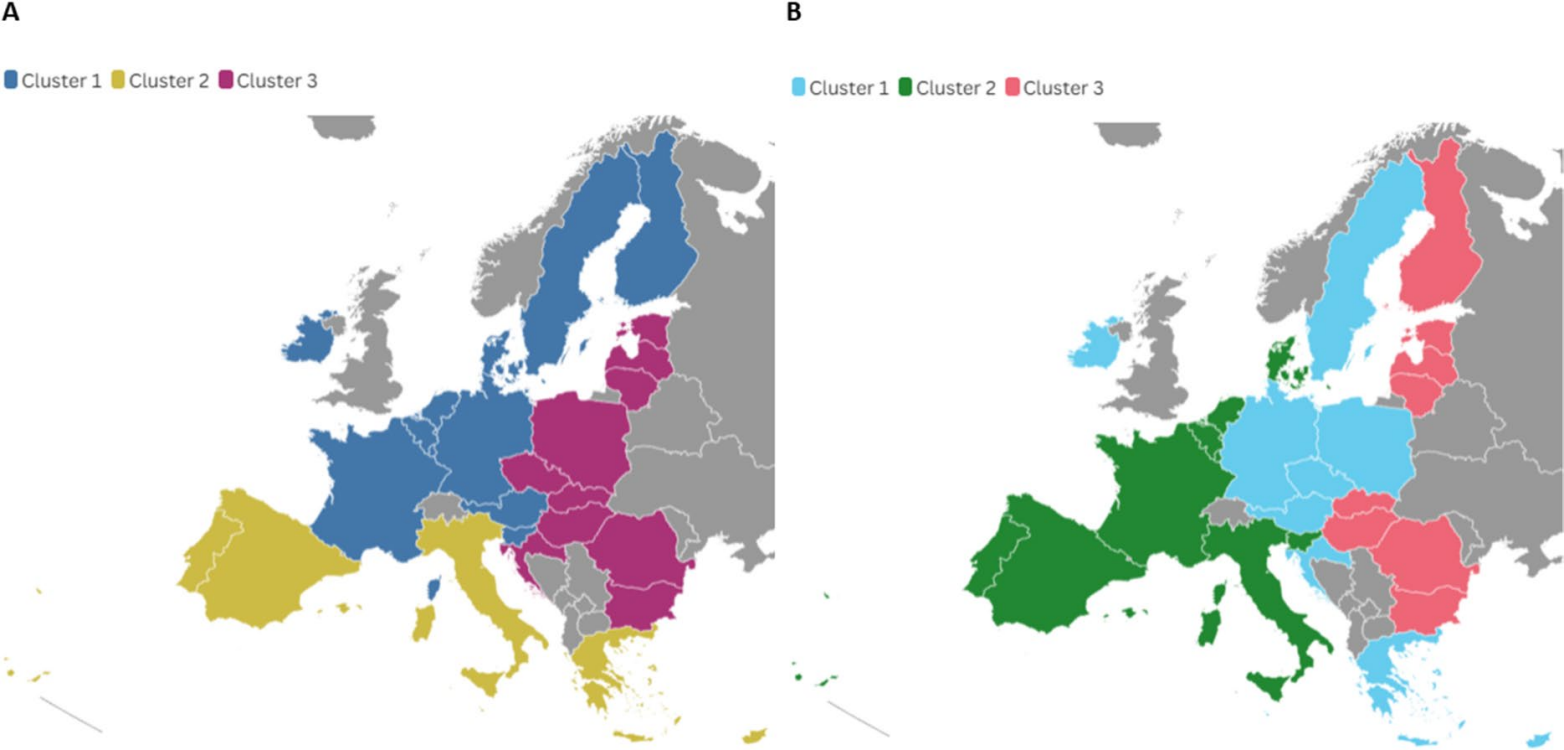
Classification of countries based on avoidable mortality indicators (**A**) and cause-specific composition of avoidable mortality (**B**)

Figure 7 demonstrates the maps of the two cluster analyses. Similarity of the two clusterings were moderate based on ARI (0.211) and NMI (0.337).

## Discussion

This study describes avoidable mortality in the EU countries in 2022 excluding deaths attributed to COVID-19. We analyzed preventable and treatable mortality rates, the ratio to each other, as well as the male-to-female ratio of avoidable mortality rates ratio as well. Then, we used two complementary hierarchical cluster analyses to explore patterns of avoidable mortality across the 27 EU member states in 2022.

The first analysis which was based on standardized indicators of preventable and treatable mortality, and their respective ratio, as well as the male-to-female avoidable mortality ratio, identified three clusters broadly corresponding to Western/Northern Europe (Cluster 1: low overall avoidable mortality and balanced sex gaps), Southern Europe (Cluster 2: low-to-moderate overall mortality but elevated preventable‐to‐treatable ratios and sex disparities, and Central/Eastern Europe (Cluster 3: high preventable and treatable mortality with pronounced male disadvantage. The second analysis which was based on the percentage distribution of causes of avoidable deaths also yielded three clusters: one with a mixed burden (balanced shares of cancer, cardiovascular, and other causes), one dominated by cardiovascular and substance‐related deaths, and one dominated by cancer and injury‐related mortality.

Although the two cluster analyses were based on different metrics, certain patterns of alignment and divergence emerged. Many Central and Eastern European countries (Bulgaria, Estonia, Hungary, Latvia, Lithuania, Romania, and Slovakia) were consistently classified in Cluster 3, highlighting both high mortality levels and a cause distribution often dominated by preventable deaths. This pattern suggests the coexistence of insufficient public health interventions targeting primary prevention and severe limitations in timely and/or effective healthcare. The prominence of cardiovascular and alcohol-related causes in the cause-based clusters supports this interpretation. Sustained population-level control of risk factors (such as hypertension, tobacco use, diet and alcohol consumption) and improvements across the continuum of care, from primary prevention to acute cardiac services are required to decrease not only cardiovascular mortality, but also cancer- and alcohol-related mortality [25–27].

These countries also exhibited a high male-to-female ratio of avoidable mortality rates. Although the underlying drivers were not investigated in our study, however, evidence shows that male populations in these regions have substantially higher prevalence of behavioral risk factors (e.g., smoking, alcohol consumption, unhealthy diet) [28–30]. These factors likely contribute to the high preventable mortality rates, and specifically to the dominance of avoidable cardiovascular and alcohol-related mortality [30–32], as observed in Cluster 3 of the second cluster analysis. However, it is also worth noting that smoking prevalence among women has been rising in several countries, warranting special attention in future public health strategies [33].

Several countries, including Austria, Germany, and Sweden, consistently appeared in Cluster 1 in both analyses, suggesting that they have both favorable mortality levels and a typical cause distribution. Italy, Spain, Portugal were placed in Cluster 2 in both analyses, representing favorable avoidable mortality levels with cancer-related cause dominance in this region. This pattern is typically observed in high-income countries, reflecting successes in cardiovascular disease prevention, thereby shifting the relative burden towards cancers [34]. High cancer shares may reflect aging-related cancer incidence, lifestyle exposures, and remaining challenges in early detection [35–38].

Finland presented a favorable overall avoidable mortality pattern (belonging to Cluster 1 in the first cluster analysis) but the cause-specific analysis revealed cardiovascular, alcohol- and drug-related causes to be the main ones, similarly to Central and Eastern European countries. This reflects the historical cardiovascular burden, which has declined, but still shapes the distribution of causes [39].

Countries such as Belgium, Denmark, France, Luxembourg, the Netherlands, and Slovenia were in Cluster 1 based on avoidable mortality indicators but were classified into Cluster 2 in the cause-based analysis. This indicates that their favorable overall mortality rates are associated with high proportion of cancer related avoidable mortality. Cyprus, Greece and Malta had an equal distribution of preventable and treatable mortality, and also cancer and cardiovascular deaths, but with sex inequalities. Croatia, Czechia, Poland experienced a high rate of avoidable mortality, but in cause specific pattern they are becoming similar to western countries.

Overall, while the clusters do not align perfectly, the two approaches complement each other. The first approach identifies countries with similar levels and patterns of avoidable mortality, while the second approach highlights similarities in the composition of health threats. Their combination offers a more nuanced understanding of regional health profiles within the European Union.

Our findings align with earlier work documenting persistent East–West mortality gradients in Europe [40], driven by cardiovascular diseases and alcohol‐related harm in post‐socialist states [41], and with studies showing that Western Europe has shifted toward a cancer‐dominant avoidable mortality pattern as cardiovascular deaths decline [42]. The large male-to-female avoidable mortality ratios in Central/Eastern and Southern clusters echo the sex disparities reported by previous studies [43].

The divergent profiles identified in our study call for tailored public health strategies. Western European countries should consolidate gains by focusing on emerging challenges, such as cancer screening uptake, injury prevention, and addressing residual sex gaps. Eastern European countries require intensified primary prevention of cardiovascular and substance‐related risks (e.g., harmonized alcohol policies, tobacco taxes) alongside strengthening healthcare access and quality for treatable conditions, especially among men. Southern countries would benefit from reinforcing preventive services particularly targeting modifiable risk factors and ensuring equitable healthcare utilization to close sex differentials. Cross-cluster learning platforms within the EU could facilitate exchange of best practices, for instance, adapting Nordic alcohol‐harm reduction strategies or Mediterranean lifestyle promotion in Central and Eastern Europe [44]. Furthermore, injuries being the third most common cause of avoidable mortality in the EU, highlights the need for injury prevention, especially in aging populations [45].

### Strengths and limitations

A major strength is the use of two orthogonal clustering approaches that together offer a nuanced portrait of avoidable mortality. The analyses rely on harmonized, age-standardized Eurostat data, ensuring comparability. However, limitations include the ecological design, which precludes causal inference at the individual level, and potential misclassification in cause‐of‐death registration. Although we excluded deaths directly attributed to COVID-19, indirect pandemic effects, such as delayed care, reduced screening uptake, elective surgery backlogs, and increases in mental-health and substance-use problems, may have influenced avoidable mortality in 2022. Furthermore, the cross-sectional design of our study does not allow us to examine temporal changes in avoidable mortality patterns. The use of cause-specific shares in the second cluster analysis allowed us to characterize the epidemiological composition of avoidable mortality independently of its total level. Although this approach is informative, future research could enhance robustness by applying log-ratio transformations or alternative compositional clustering techniques, which would help to address the inherent constraints of compositional data. Finally, clustering results depend on methodological choices (e.g., Ward’s method, number of clusters), though convergence across analyses supports robustness.

## Conclusions

This study demonstrates that avoidable mortality in the EU exhibits distinct regional patterns, both in overall burden and in underlying cause structure. Recognizing these patterns is crucial for designing targeted interventions that can most effectively reduce premature deaths and promote healthy aging. Our findings highlight clear priorities for action, including strengthening preventive and screening services, reducing sex disparities in healthcare use, and fostering cross-country exchange of effective public-health strategies. Implementing these targeted measures may help accelerate progress toward reducing avoidable mortality across the EU. Future research should explore temporal trends to assess whether clusters are converging and evaluate which policy measures most effectively shift countries toward lower mortality profiles. Reducing avoidable mortality is not only a benchmark of healthcare performance but also a cornerstone of Europe’s healthy aging agenda. In alignment with the WHO Decade of Healthy Ageing (2021–2030), our findings support the adaptation of national and EU-level strategies that address the determinants of avoidable mortality—including cardiovascular, cancer, and alcohol-related risks—through integrated prevention and equitable access to care. Strengthening the synergy between WHO initiatives and EU frameworks could accelerate progress toward reducing avoidable deaths, closing the East–West health divide, and fostering equitable longevity across generations in Europe. In this context, collaborative networks such as the WHO Collaborating Centre on Healthy Ageing and the European Public Health Association (EUPHA) can play pivotal roles in translating scientific evidence into coordinated public health action. By harmonizing national strategies, facilitating knowledge exchange, and promoting coherent, cross-border policies, these networks support the shared vision of enabling all Europeans to live longer, healthier, and more equitable lives.

## Data Availability

The avoidable/preventable/treatable mortality data used in this study are publicly available from the Eurostat online database (https://ec.europa.eu/eurostat). The processed country-level datasets and analysis scripts are available from the corresponding authors upon request.

## References

[1] Eurostat. Population structure and ageing. 03/07/2025]; Available from: https://ec.europa.eu/eurostat/statistics-explained/index.php?title=Population_structure_and_ageing.

[2] OECD/European Commission, *Health at a Glance: Europe 2024: State of Health in the EU Cycle*. 2024: Paris.

[3] Garmany A, Terzic A. Global healthspan-lifespan gaps among 183 World Health Organization member states. JAMA Netw Open. 2024;7(12):e2450241. 39661386 10.1001/jamanetworkopen.2024.50241 PMC11635540

[4] Eurostat. *EU life expectancy at birth 80.6 years in 2022*. 2024 23/09/2025]; Available from: https://ec.europa.eu/eurostat/web/products-eurostat-news/w/ddn-20240314-1#:~:text=In%202022%2C%20the%20life%20expectancy%20at%20birth,years%20(up%203.7%20years%20compared%20with%202002).

[5] Kuan V, et al. Data-driven identification of ageing-related diseases from electronic health records. Sci Rep. 2021;11(1):2938. 33536532 10.1038/s41598-021-82459-y PMC7859412

[6] Eurostat. Causes of death statistics. 2025 23/09/2025]; Available from: https://ec.europa.eu/eurostat/statistics-explained/index.php?title=Causes_of_death_statistics#:~:text=The%20leading%20causes%20of%20death%20were%20diseases,which%20accounted%20for%207%25%20and%206%25%2C%20respectively.

[7] Khan HTA, Addo KM, Findlay H. Public health challenges and responses to the growing ageing populations. Public Health Challenges. 2024;3(3):e213. 40496520 10.1002/puh2.213 PMC12039680

[8] Association of Schools & Programs of Public Health, Healthy Longevity: Public Health’s Next Frontier, a Framework for Research, Education, Practice, and Policy. 2025. https://aspph-webassets.s3.us-east-1.amazonaws.com/ASPPH-Healthy-Longevity.pdf. Accessed 24 Jul 2025.

[9] Nolte E, McKee M. Does health care save lives? Avoidable mortality revisited. London: The Nuffield Trust. 2004.

[10] Eurostat and OECD, Avoidable mortality: OECD/Eurostat lists of preventable and treatable causes of death (January 2022 version). 2022. Publications Office of the European Union. https://www.oecd.org/content/dam/oecd/en/data/datasets/oecd-health-statistics/avoidable-mortality-2019-joint-oecd-eurostat-list-preventable-treatable-causes-of-death.pdf. Accessed 15 June 2025.

[11] Health-Europe TL. Securing the future of Europe’s ageing population by 2050. Lancet Regional Health-Europe. 2023;1(35):100807. 10.1016/j.lanepe.2023.100807 PMC10730304 38115962

[12] Xi JY, Lin X, Hao YT. Measurement and projection of the burden of disease attributable to population aging in 188 countries, 1990–2050: a population-based study. J Glob Health. 2022;12:04093. 36259226 10.7189/jogh.12.04093 PMC9579832

[13] Hrzic R, Vogt T. The contribution of avoidable mortality to life expectancy differences and lifespan disparities in the European Union: a population-based study. Lancet Reg Health. 2024;46:101042. 10.1016/j.lanepe.2024.101042 PMC11402299 39286330

[14] Skybová D, et al. Risk of chronic diseases limiting longevity and healthy aging by lifestyle and socio-economic factors during the life-course - a narrative review. Medyc Pr. 2021;72(5):535–48. 10.13075/mp.5893.01139 34664558

[15] Cherla A, et al. Trends in avoidable mortality from cardiovascular diseases in the European Union, 1995–2020: a retrospective secondary data analysis. Lancet Reg Health. 2024;47:101079. 10.1016/j.lanepe.2024.101079 PMC11470399 39397877

[16] Onofrei M, et al. Government health expenditure and public health outcomes: a comparative study among EU developing countries. Int J Environ Res Public Health. 2021. 10.3390/ijerph182010725. 34682472 10.3390/ijerph182010725 PMC8535729

[17] Santos JV, et al. The state of health in the European Union (EU-27) in 2019: a systematic analysis for the Global Burden of Disease study 2019. BMC Public Health. 2024. 10.1186/s12889-024-18529-3. 38778362 10.1186/s12889-024-18529-3 PMC11110444

[18] Stirbu I, et al. Educational inequalities in avoidable mortality in Europe. J Epidemiol Community Health. 2010;64(10):913–20. 19833607 10.1136/jech.2008.081737

[19] Khoshmaram N, et al. Strategies and challenges for maintaining the continuity of essential health services during a pandemic: a scoping review. BMC Health Serv Res. 2025;25(1):691. 40361091 10.1186/s12913-025-12812-8 PMC12077003

[20] Khan Y, et al. Evaluating the health and health economic impact of the COVID-19 pandemic on delayed cancer care in Belgium: a Markov model study protocol. PLoS ONE. 2023;18(10):e0288777. 37903130 10.1371/journal.pone.0288777 PMC10615261

[21] Arsenault C, et al. COVID-19 and resilience of healthcare systems in ten countries. Nat Med. 2022;28(6):1314–24. 35288697 10.1038/s41591-022-01750-1 PMC9205770

[22] Allahqoli L, et al. Impact of COVID-19 on cancer screening: a global perspective. Curr Opin Support Palliat Care. 2022;16(3):102–9. 35862881 10.1097/SPC.0000000000000602 PMC9451605

[23] Roszko-Wojtowicz E, Przybysz K, Stanimir A. Unequal ageing: the quality of life of senior citizens in the EU before and after COVID-19. A multidimensional approach. Front Public Health. 2025;13:1506006. 39944069 10.3389/fpubh.2025.1506006 PMC11815594

[24] Eurostat, Treatable and preventable mortality of residents by cause and sex. 10.2908/HLTH_CD_APR

[25] Mensah GA, et al. Decline in cardiovascular mortality: possible causes and implications. Circ Res. 2017;120(2):366–80. 28104770 10.1161/CIRCRESAHA.116.309115 PMC5268076

[26] Yusuf S, et al. Modifiable risk factors, cardiovascular disease, and mortality in 155 722 individuals from 21 high-income, middle-income, and low-income countries (PURE): a prospective cohort study. Lancet. 2020;395(10226):795–808. 31492503 10.1016/S0140-6736(19)32008-2 PMC8006904

[27] Gallucci G, et al. Cardiovascular risk of smoking and benefits of smoking cessation. J Thorac Dis. 2020;12(7):3866–76. 32802468 10.21037/jtd.2020.02.47 PMC7399440

[28] Eurostat. Tobacco consumption statistics. 18/11/2025]; Available from: https://ec.europa.eu/eurostat/statistics-explained/index.php?title=Tobacco_consumption_statistics.

[29] Eurostat. Alcohol consumption statistics. 18/11/2025]; Available from: https://ec.europa.eu/eurostat/statistics-explained/index.php?title=Alcohol_consumption_statistics.

[30] Stefler D, et al. Traditional Eastern European diet and mortality: prospective evidence from the HAPIEE study. Eur J Nutr. 2021;60(2):1091–100. 32613328 10.1007/s00394-020-02319-9 PMC7900332

[31] Jasilionis D, Leon DA, Pechholdova M. Impact of alcohol on mortality in Eastern Europe: trends and policy responses. Drug Alcohol Rev. 2020;39(7):785–9. 33222293 10.1111/dar.13167

[32] Stefler D, et al. Smoking and mortality in Eastern Europe: results from the PrivMort retrospective cohort study of 177 376 individuals. Nicotine Tob Res. 2018;20(6):749–54. 28575492 10.1093/ntr/ntx122

[33] Janssen F, El Gewily S, Bardoutsos A. Smoking epidemic in Europe in the 21st century. Tob Control. 2021;30(5):523–9. 32769210 10.1136/tobaccocontrol-2020-055658 PMC8403059

[34] Mahase E. Cancer overtakes CVD to become leading cause of death in high income countries. BMJ. 2019;366:l5368. 31481521 10.1136/bmj.l5368

[35] Laconi E, Marongiu F, DeGregori J. Cancer as a disease of old age: changing mutational and microenvironmental landscapes. Br J Cancer. 2020;122(7):943–52. 32042067 10.1038/s41416-019-0721-1 PMC7109142

[36] Fekete M, et al. Geroscience and pathology: a new frontier in understanding age-related diseases. Pathol Oncol Res. 2024;30:1611623. 38463143 10.3389/pore.2024.1611623 PMC10922957

[37] Perez DG, Loprinzi C, Ruddy KJ. Lifestyle factors can lead to multiple cancers over a lifetime-here we go again. JAMA Oncol. 2021;7(4):505–6. 33351067 10.1001/jamaoncol.2020.7360

[38] Crosby D, et al. Early detection of cancer. Science. 2022;375(6586):eaay9040. 35298272 10.1126/science.aay9040

[39] Borodulin K, et al. Forty-year trends in cardiovascular risk factors in Finland. Eur J Public Health. 2015;25(3):539–46. 25422363 10.1093/eurpub/cku174

[40] Muller-Nordhorn J, et al. An update on regional variation in cardiovascular mortality within Europe. Eur Heart J. 2008;29(10):1316–26. 18256043 10.1093/eurheartj/ehm604

[41] Glushkova N, Semenova Y, Sarria-Santamera A. Editorial: Public health challenges in post-Soviet countries during and beyond COVID-19. Front Public Health. 2023;11:1290910. 37886052 10.3389/fpubh.2023.1290910 PMC10598333

[42] Cabasag CJ, et al. The preventability of cancer in Europe: a quantitative assessment of avoidable cancer cases across 17 cancer sites and 38 countries in 2020. Eur J Cancer. 2022;177:15–24. 36323048 10.1016/j.ejca.2022.09.030

[43] Crimmins EM, Kim JK, Sole-Auro A. Gender differences in health: results from SHARE, ELSA and HRS. Eur J Public Health. 2011;21(1):81–91. 20237171 10.1093/eurpub/ckq022 PMC3023013

[44] Ungvari Z, et al. Adherence to the Mediterranean diet and its protective effects against colorectal cancer: a meta-analysis of 26 studies with 2,217,404 participants. Geroscience. 2025;47(1):1105–21. 39090501 10.1007/s11357-024-01296-9 PMC11872821

[45] Xu Q, Ou X, Li J. The risk of falls among the aging population: a systematic review and meta-analysis. Front Public Health. 2022;10:902599. 36324472 10.3389/fpubh.2022.902599 PMC9618649

